# Spirulina Supplementation Alleviates Intense Exercise-Induced Damage and Modulates Gut Microbiota in Mice

**DOI:** 10.3390/nu17020355

**Published:** 2025-01-20

**Authors:** Chunxia Wang, Huijuan Liu, Shuyu Zhang, Chengyi Ren, Jiaming Xu, Juanjuan Chen, Haimin Chen, Wei Wu

**Affiliations:** 1 State Key Laboratory for Managing Biotic and Chemical Threats to the Quality and Safety of Agro-Products, Ningbo University, Ningbo 315211, China; 2 Collaborative Innovation Center for Zhejiang Marine High-Efficiency and Healthy Aquaculture, Ningbo University, Ningbo 315211, China

**Keywords:** *Spirulina*, antioxidant, gut microbiota, high-intensity exercise

## Abstract

Background: *Spirulina*, which are filamentous cyanobacteria, have gained significant popularity in the food industry, medicine, and aquaculture. Methods: In this study, our objective was to explore the influence of *Spirulina* on the gut microbiota and exercise capacity of mice undergoing high-intensity exercise. Twenty-four male BALB/c mice were divided into four groups, with six mice in each group. These groups included the control group (Control, in which the mice received saline gavage and were permitted free movement), the exercise group (Running, in which the mice were gavaged with the same volume of saline and subjected to a structured exercise regimen), and the *Spirulina* intervention groups (including *Spirulina* ^Low^ and *Spirulina* ^High^). In the *Spirulina* intervention groups, the mice were orally administered with *Spirulina* at doses of 100 and 300 mg/kg/day for four weeks while simultaneously participating in the exercise protocol. Results: The results illustrated that the Running group mice subjected to intense exercise exhibited reduced weight and tension, acute damage to muscle and liver tissues, oxidative stress, and an imbalance in the gut microbiota compared with that of the Control group. However, high-concentration *Spirulina* supplementation was found to increase the tensile strength of the exercise mice by 1.27 ± 0.19 fold (*p* < 0.05) and ameliorate muscle and liver damage. In the *Spirulina* ^High^ group, the levels of certain indicators related to muscle oxidative stress, including reactive oxygen species, total superoxide dismutase, and catalase, were decreased by 39 ± 5.32% (*p* < 0.01) and increased by 1.11 ± 0.17 fold and 1.19 ± 0.22 fold (*p* < 0.01) compared to the Running group. Additionally, a correlation analysis reveals that the alterations in gut microbiota induced by *Spirulina* might be associated with the indicators of tension and oxidative stress. Conclusions: Collectively, these findings point to the fact that *Spirulina* can effectively mitigate the acute damage to muscles and the liver induced by intense exercise in mice by enhancing antioxidant capacity and regulating the gut microbiota, thereby providing novel insights into the mechanism underlying the enhancement of exercise function.

## 1. Introduction

Physical exercise is commonly acknowledged as a modality for managing physical health and is also among the strategies for combating and managing diseases [1]. However, a heart rate reaching 70–90% of the maximum heart rate during high-intensity exercise, such as sprinting, heavy-load weightlifting, or competitive sports, may tend to induce oxidative injury and trigger the generation of reactive oxygen species (ROS). Consequently, this causes oxidative damage and has harmful effects on human health [2]. Fortuitously, to preclude oxidative damage by high-intensity exercise, cells have evolved intricate endogenous antioxidant defense mechanisms. These mechanisms operate by secreting a repertoire of antioxidant enzymes, such as SOD and HO-1, to counteract oxidative stress [3]. Moreover, exogenous dietary antioxidants engage in interactions with endogenous antioxidants, thereby constituting a cellular antioxidant network [4]. Therefore, the supplementation of antioxidant nutrients may present a prospective strategy for preventing and ameliorating oxidative stress-induced muscle damage in athletes.

At present, athletes routinely resort to dietary supplements to expedite the repair process and mitigate oxidative stress-induced injuries, such as muscle soreness, fatigue, and joint stiffness, that ensue after training regimens [5]. To avoid potential doping test violations [6], an increasing number of coaches, fitness trainers, and sports nutritionists are focusing on pure natural supplements [7]. These supplements, which are sourced from naturally occurring flora or fauna through non-synthetic chemical production methods, are believed to have enhanced biological accessibility and superior efficacy compared to natural extracts or synthetic compounds [8]. *Spirulina*, a bioactive photosynthetic blue–green alga rich in essential amino acids, fatty acids, tocopherol, beta carotene, polyphenols, and phycocyanin—all of which exhibit antioxidant properties—has been experimentally proven to be safe in both chronic and acute administrations in the liver, kidneys, reproductive system, and overall human physiology [9]. It is classified as ‘Generally Recognized as Safe’ (GRAS) by the U.S. FDA [10]. Additionally, *Spirulina* has been shown to regulate the intestinal microbiome and promote the growth of beneficial bacteria, such as *Bifidobacterium* and *Lactobacillus* [11].

Nevertheless, due to the stringent regulations governing competitions, athletes are extremely cautious when selecting sports supplements. Certain dietary adjuncts may inadvertently give rise to substances that are prohibited in competitive settings or even aggravate the progression of oxidative damage [12]. For instance, Oliveira et al. found that vitamins C and E can reduce oxidative stress, but they do not improve biomarkers for acute exercise-related muscle injury or soreness, nor enhance football performance in young athletes [13]. Vitamin E serves as a lipid-soluble antioxidant. However, in active individuals, it may aggravate oxidative stress due to lipid peroxidation and inflammation [14]. Resveratrol, a natural polyphenol that activates nuclear transcription factor Nrf2 to exert antioxidant effects, has been tested for its ability to reduce muscle fatigue [15]. Nevertheless, it has been proven to increase oxidative damage in skeletal muscles after exercise [16]. Although it has been included in several trials on human subjects, mainly to evaluate its effectiveness in alleviating muscle fatigue, the results have indicated that resveratrol exacerbates oxidative stress damage in skeletal muscles caused by exercise training. Additionally, the long-term safety of another common supplement, phosphocreatine, remains to be studied.

Our previous research has confirmed that the *Spirulina* supplement can inhibit the decrease in the ratio of leukocytes and monocytes in young athletes, which is induced by intense long-duration exercise [17]. Meanwhile, Mahmood et al. have further demonstrated the crucial role of the antioxidant property of *Spirulina* in protecting against Cyclosporine-induced oxidative stress [18]. However, to date, there remains a dearth of reports concerning antioxidant levels, the amelioration of oxidative damage, and regulatory influence on the gut microbiota exerted by *Spirulina* within sports populations. In the present study, a model of running exhaustion was constructed using BALB/c mice to comprehensively compare the impacts of diverse doses of *Spirulina* on an array of parameters in mice subjected to high-intensity exercise. These parameters encompass weight, pathological changes in muscle and liver tissue, alterations in oxidative stress levels, exercise capacity, and modifications within the gut microbiota. Our investigations established that *Spirulina* exhibits potent antioxidant properties, thereby effectively mitigating muscle injury, augmenting tensile strength, and precisely shaping the functions and composition of the gut microbiota in mice experiencing running exhaustion. This modulation not only influences the relative abundances of various microbial taxa but also their metabolic activities and interactions, thereby contributing to the overall physiological homeostasis of the host organism during and after strenuous exercise.

## 2. Methods and Materials

### 2.1. Animals and Treatment

All BALB/c mice were procured from Slack Corporation (Jiangsu, China) and subsequently accommodated within the Animal Center of Ningbo University (Ningbo, China). The laboratory mice were maintained under rigorously controlled environmental conditions, with the temperature precisely regulated at 21 ± 2 °C and the humidity maintained within the range of 50 ± 10%. A 12 h light–dark cycle was implemented to mimic natural diurnal rhythms. Mice feed was purchased from Beijing Ke’ao xieli Feed Co., Ltd. (Beijing, China) without any added antibiotics, insect repellents, preservatives, pigment, growth-promoting agents, hormones, and food additives, including *Spirulina*. The nutrition components of the experimental animal feed conform to the GB 14924.3-2023 standards [19] (Table S1). All experimental protocols adhered stringently to the guidelines stipulated by the National Institutes of Health for the ethical care and utilization of experimental animals. Notably, all animal-based experimental undertakings were granted formal approval by the Ethics Committee for Animal Use and Protection of the Ningbo University Health Science Center (Approval No. NBU 23165).

For experimental categorization, the experimental design encompassed three distinct groups. The control group (designated as Control) received saline gavage and was permitted unrestricted movement. The exercise group (termed Running) was subjected to saline gavage in conjunction with a structured exercise regimen. The *Spirulina* intervention group was further subdivided into two subgroups: *Spirulina* ^Low^ and *Spirulina* ^High^. In these subgroups, BALB/c mice were orally administered with sterilized *Spirulina* powder at concentrations of 100 and 300 mg/kg, respectively, while simultaneously engaging in the exercise protocol. Each group consisted of six mice. The daily administration of gastric gavage was scheduled precisely at 8 am, and the exercise and training sessions were systematically conducted at 8 pm every night.

The *Spirulina* powder employed in this experiment, sourced from Yunnan Chenghaibao Company in Yunnan Province, China, has been duly certified as safe for use in this research context.

### 2.2. Sports Training Plan

Male BALB/c mice, aged 10 weeks, were randomly stratified into four discrete cohorts: (1) the free-moving control group (Control, mice were gavaged with saline), which served as the baseline comparator; (2) the treadmill exercise group (Running, mice were gavaged with the same volume of saline), designed to experience exercise-induced physiological perturbations; (3) the 100 mg/kg *Spirulina* intervention group (*Spirulina* ^Low^); and (4) the 300 mg/kg *Spirulina* intervention group (*Spirulina* ^High^). The concentrations of *Spirulina* in cohorts (3) and (4) received by oral administrations were selected based on preliminary experiments in a related study [17,20]. Groups (3) and (4) of mice were subjected to a meticulously designed 4-week running exercise regimen, entailing 4 days of activity per week, with each session lasting 30 min at a constant speed of 12 m/min on a 20° inclined treadmill (model ZH-PT, Zhenghua, Guangdong, China). The exercise plan was selected based on previous high-intensity exercise training studies [21] and made some minor changes. This exercise model bears similarities to the physiological load endured by athletes during competitions or specific training periods, such as long-distance running, sprinting, soccer, basketball, and so on.

Groups of mice underwent an acclimatization phase, involving 3 consecutive days of moderate treadmill running at 10 m/min for 5 min daily. Subsequently, the mice initiated the formal test, commencing at 10 m/min for 5 min, followed by a progressive increase in speed from 10 m/min to 16 m/min until the point of exhaustion. All experimental procedures involving mice were subject to and received approval from the Animal Ethics Committee of Ningbo University (Approval No. NBU230739), ensuring strict adherence to ethical standards in animal research.

### 2.3. Tissue Histology

Collect the muscles, liver, and gut in each animal, wash the gut employing ice-cold sterile PBS, and aseptically collect the colon samples for gut microbiota analysis. Fix 1 cm muscle and liver tissue slices in saline containing 10% formaldehyde solution for staining with hematoxylin-eosin stain (H&E). The degree of histologic damage was evaluated in a blinded fashion by expert histologists.

### 2.4. Biochemical Analysis Related to Oxidation Indicators

Evaluate the oxidative stress changes in the mouse muscles of the Control, Running, *Spirulina* ^Low^, and *Spirulina* ^High^ groups by measuring ROS, total superoxide dismutase (T-SOD), and catalase (CAT) using EDTA. Operate according to the instructions of the Mouse ROS ELISA detection kit (Shanghai Yuanju Biotechnology Center, Shanghai, China), T-SOD assay kit (Nanjing Jiancheng, Nanjing, China), and CAT assay kit (Suzhou Greasy Biotechnology Co., Ltd., Suzhou, China).

### 2.5. Bioinformatics and Analysis of Sequencing Data

According to the manufacturer’s instructions, genomic DNA was obtained from fecal samples via the Powersoil DNA kit (Qiagen, Hilden, Germany) [22] The level, clarity, and completeness of the fecal DNA were measured in each cohort of mice. Utilizing the derived DNA as a template, the primer pairs F: AGRGTTGATYNTGGCTCAG and R: TASGGHTACCTTGTTASGACTT amplify DNA [23]. The 5’ end of the primer includes the universal sequence of the Illumina linker. Purify the obtained PCR products and introduce them into specific tag sequences compatible with the Illumina platform using high-fidelity PCR to construct the final complete library. After evaluating the quality of the library, the V3–V4 hypervariable region of the 16S rRNA gene was amplified by a two-step PCR reaction and then sequenced by an Illumina Miseq Sequencing platform to generate raw readings [24]. Then, from these data, sequences with a shared similarity of ≥ 97% were aggregated into OTUs, which would avoid the misclassification of sequences belonging to the same species into different OTUs, allowing for a relatively reasonable classification of microbial communities, even with some errors. For comparison of the composition of gut microbiota OTUs across each group, a Venn picture was designed based on the R package (version 3.1.0). To identify inter- and intra-group differences, non-metric multi-dimensional scaling (NMDS) was used for sample analysis, which was better able to capture the underlying patterns and relationships in the data. Then, use ChiPlot (https://www.chiplot.online/ Date is 28 November 2024.). Use online analysis tools to create stacked bar charts and analyze changes in gut microbes across the phylum and genus levels. When using the Python LEfSe package(http://huttenhower.sph.harvard.edu/lefse/ accessed on 16 February 2023) for analysis, linear discriminant analysis (LDA) is then employed to assess the magnitude of the impact of each component (species) abundance on the differential effect.

### 2.6. Statistical Examination

The figures for weight, tensile strength, and oxidative stress-related indicators are expressed as the mean ± standard deviation. A statistical analysis was conducted using one-way ANOVA and Bartlett’s test (SPSS 12.0, North Chicago, IL, USA). Compared with the control group, all results were regarded as statistically significant at * *p* < 0.05 and crucial at ** *p* < 0.01. Compared with the Running group, all results were deemed statistically remarkable at ^#^ *p* < 0.05 and enormously remarkable at ^##^ *p* < 0.01.

## 3. Results

### 3.1. Consuming Spirulina Can Increase the Weight and Tension of Exercise Mice and Improve High-Intensity Exercise-Induced Muscle and Liver Damage

**Figure 1:**
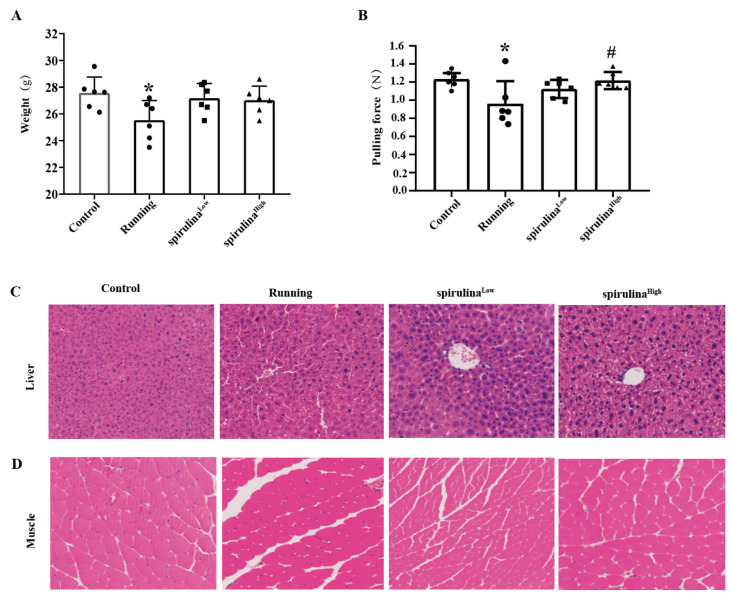
*Spirulina* was found to affect body weight, tensile strength, liver, and muscle in experimental mice. Mouse weight (**A**); mouse tensile strength (**B**); H&E staining of mouse liver and muscle treated with *Spirulina* (**C**,**D**). The results were shown as mean ± standard deviation Compared with the Control group, * *p* < 0.05, ^#^ *p* < 0.05, as opposed to the Control group. Abbreviations: sedentary group (Control), exercise group (Running), receiving low concentrations of *Spirulina* group (*Spirulina* ^Low^), receiving high concentrations of *Spirulina* group (*Spirulina* ^High^).

As depicted in Figure 1A, a statistically significant reduction in both weight and tensile strength was observed in the Running group relative to the Control group (*p* < 0.05). In contrast, when compared with the Running group, both low and high concentrations of *Spirulina* elicited a rise in the body weight of high-intensity exercise mice, albeit without attaining statistical significance (*p* > 0.05). Notably, treatment with high-concentration *Spirulina* engendered a 1.27 ± 0.19-fold augmentation in tensile strength to the Running group (*p* < 0.05; Figure 1B).

To further probe the impact of *Spirulina* on tissue damage in high-intensity exercise mice, the hematoxylin and eosin (H&E) staining technique was employed. In the control group, muscle fibers were found to be orderly arranged, with a distinct and uniformly distributed nuclear structure. Conversely, in the Running group, an expansion of the inter-fiber spaces was evident, accompanied by a disarray of the muscle fibers and vacuolar alterations within the muscle cells. Upon intervention with high concentrations of *Spirulina*, the damaged musculature of exercise mice underwent a gradual restoration process mediated by uniform fibers. This led to a more favorable preservation of muscle fiber dimensions post-injury and a concomitant substantial diminution in muscle fibrosis compared with that of the Running group (Figure 1C).

An analysis of pathological tissue sections of the liver in the exercise mice divulged that the Running group exhibited an aberrant liver tissue architecture, characterized by extensive damage across the entire liver lobule and a disrupted arrangement of liver cell cords. In contrast, within the *Spirulina* ^High^ group, the liver cells were more tightly packed and arrayed radially around the central vein compared with the Running group (Figure 1D). These findings unequivocally suggest that *Spirulina* is capable of mitigating the damage inflicted upon liver and muscle tissues during high-intensity exercise.

### 3.2. Spirulina Reduces Muscle Oxidative Damage in High-Intensity Exercise Mice

**Figure 2:**
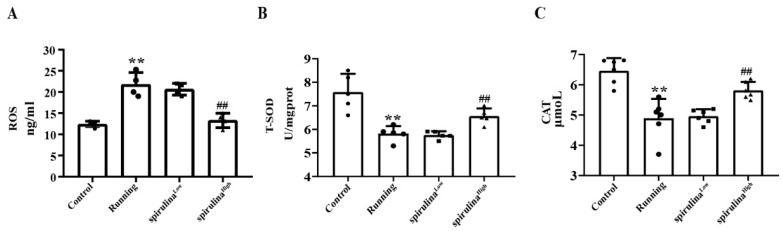
Muscle antioxidant index ((**A**) representative active oxygen, (**B**) representative total superoxide dismutase, (**C**) representative catalase). ** *p* < 0.01; compared with the Running group and ^##^ *p* < 0.01, compared with the Running group.

Intense physical exercise has the potential to enhance the generation of free radicals, thereby instigating oxidative stress responses within the organism [25]. In light of this, we undertook a more comprehensive examination of alterations in specific oxidative markers within the mouse serum. As illustrated in Figure 2A, in comparison to the control cohort, strenuous exercise conspicuously augmented the production of reactive oxygen species (ROS) while concomitantly diminishing the levels of total superoxide dismutase (T-SOD) and catalase (CAT) to a statistically significant degree (*p* < 0.01).

Nevertheless, the supplementation of *Spirulina* effectively reversed the perturbations in the oxidative stress levels precipitated by high-intensity exercise. Notably, the administration of high-concentration *Spirulina* led to a remarkable 39 ± 5.32% reduction in ROS levels (*p* < 0.01). In contrast, when contrasted with the Running group, both the levels of T-SOD and CAT in the *Spirulina* ^High^ group were markedly elevated by approximately 1.11 ± 0.17 fold and 1.19 ± 0.22 times, individually (*p* < 0.01; Figure 2B,C). These findings suggest the potential of *Spirulina* to possess strong antioxidant capacity in the context of exercise-induced oxidative stress.

### 3.3. Spirulina Impacts the Variety of Gut Microbiota

The accumulation of reactive oxygen species (ROS) exerts a profound influence on the equilibrium of the gut microbiota, culminating in a diminution of beneficial bacterial populations and a concurrent augmentation of harmful bacteria. This bacterial imbalance constitutes a pivotal determinant impacting both the antioxidant endogenous defense system and physical exercise capacity [26]. In this regard, we embarked upon a more in-depth exploration of the ramifications of *Spirulina,* involving the intestinal flora of high-intensity exercise mice and employing 16S rRNA sequencing technology.

**Figure 3:**
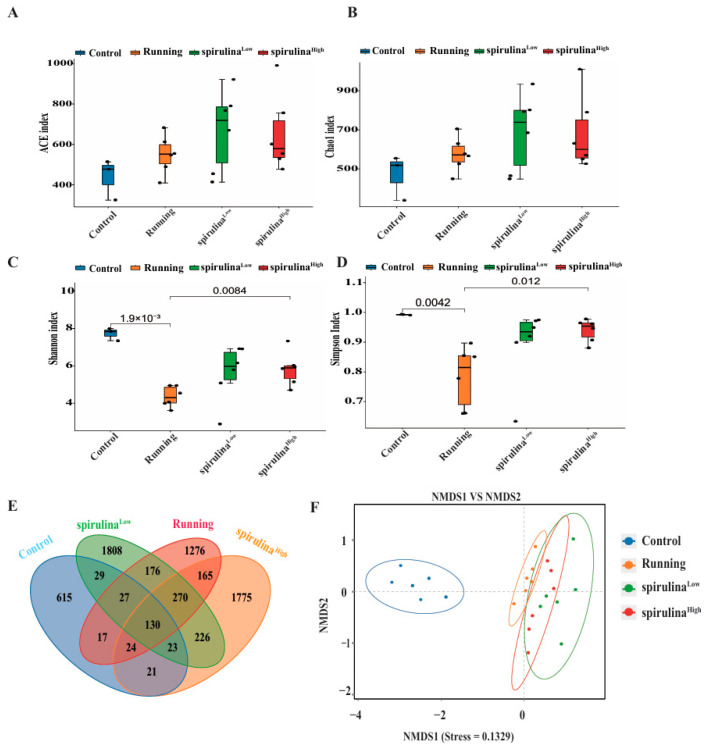
*Spirulina* alters the makeup of the microbial community in the mouse gut. Estimate the total bacterial load and alpha diversity in the gut using Ace (**A**), Chao 1 (**B**), Shannon (**C**), and Simpson (**D**) indices. *Spirulina* regulates the structure of gut microbiota. Analytic Venn diagram representation of unique/collaborative OTUs in the gut microbiota of test mice (**E**). Gastrointestinal microbiota NMDS scoring chart (**F**).

In comparison to the Control group, the Chao 1 and abundance-based coverage estimation (ACE) indices within the Running group evinced no statistically significant disparity in microbial richness. However, the Shannon index and the Simpson index manifested a marked decline (Figure 3A–D). In contrast, when juxtaposed with the Running group, the Shannon and Simpson indices of intestinal flora composition in both the *Spirulina* ^High^ and *Spirulina* ^Low^ groups of mice exhibited a substantial increase. This augmentation attests to the capacity of *Spirulina* supplementation to enhance the diversity and richness of the microbiota in high-intensity exercise mice.

A Venn diagram was created using the identified operational taxonomic units (OTUs). Figure 3E shows that each group of mice had its unique OTUs, with the running group having more OTUs than the control group. In total, there were 130 OTUs across the four groups, with the following unique OTUs: 615 in the Control group, 1276 in the Running group, 1808 in the *Spirulina* ^Low^ group, and 1775 in the *Spirulina* ^High^ group. The Control and Running groups shared 198 OTUs, while the Running and *Spirulina* ^High^ groups shared 589 OTUs. Non-metric multidimensional scaling (NMDS) analysis based on OTUs clearly showed differences between and within the groups. The Control and Running groups were notably separated. Additionally, the Running group was also distantly spaced from both the *Spirulina* ^Low^ and *Spirulina* ^High^ groups, suggesting differences in gut bacterial composition between the Running group and the *Spirulina* groups (Figure 3F).

### 3.4. Spirulina Alters Colonic Microbiota Composition in High-Intensity Exercise Mice

**Figure 4:**
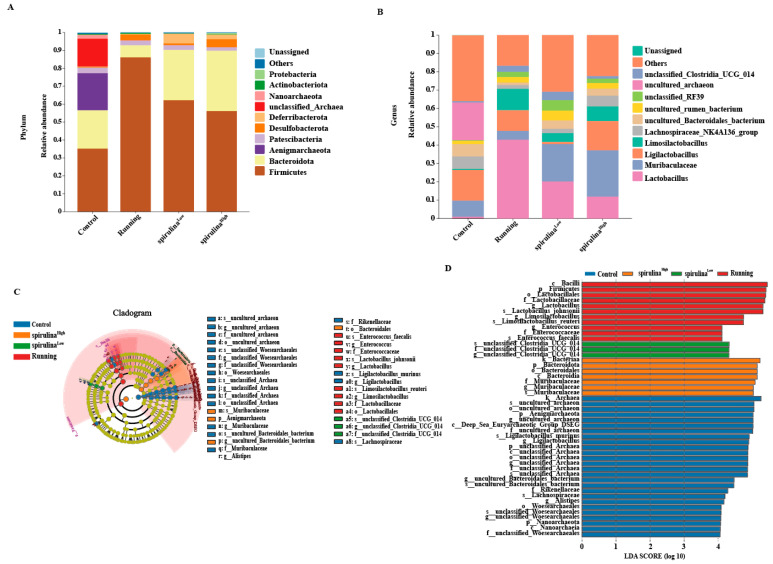
The reaction of intestinal flora to the level of phylum (**A**) and genus (**B**) to *Spirulina*. Comparison of LEfSe of gut microbiota among Control, Running, *Spirulina* ^Low^, and *Spirulina* ^High^ groups (**C**). Bar chart of LDA value distribution of *Spirulina* on mouse gut microbiota (**D**).

To comprehensively decipher the specific bacterial taxa influenced by *Spirulina*, we conducted an exhaustive analysis of the gut microbiota composition. At the phylum level, relative to the control group, the abundances of Bacteroidota and Deferribacter in the Running group were diminished by 68% and 16%, respectively. In contrast, the abundances of Firmicutes and Desulfobacterota in the Running group witnessed a remarkable elevation of 1.44 and 11.26 fold, respectively (Figure 4A). Intriguingly, in comparison with the Running group, *Spirulina* treatment in both the *Spirulina* ^Low^ and *Spirulina* ^High^ groups engendered an augmentation in the abundances of Bacteroidota and Deferribacter, while concurrently reducing the abundance of Firmicutes. Notably, as the concentration of *Spirulina* escalated, the abundance of Firmicutes in the murine gut declined significantly by 87% in a concentration-dependent manner (Figure 4A).

At the genus level, it is worthy of note that the relative abundances of *Lactobacillus* and *Limosilactobacillus* experienced a substantial increase by 40.48 and 17.05 fold, respectively. However, the relative abundances of *Ligilactobacillus*, *Lachnospiraceae_NK4A136_group*, and *Muribaculaceae* in the Running group were curtailed by 32.96%, 69.96%, and 45.16%, respectively, when contrasted with the Control group. Conversely, following *Spirulina* intervention, the abundance of *Lactobacillus* decreased, whereas the abundances of *Muribaculaceae* and the *Lachnospiraceae_NK4A136_group* augmented in a dose-dependent fashion compared with that of the Running group. Specifically, in the *Spirulina* ^High^ group, the amounts of *Muribaculaceae* and the *Lachnospiraceae_NK4A136_group* were elevated by 5.24 and 2.88 fold, respectively, while the abundance of *Lactobacillus* was reduced by 52.83% relative to the Running group (Figure 4B).

To precisely identify the specific bacteria affiliated with the Control, Running, *Spirulina* ^Low^, and *Spirulina* ^High^ groups, we implemented LEfSe (linear discriminant analysis effect size) and LDA (linear discriminant analysis) techniques. These analyses were designed to pinpoint the core taxa most apt to elucidate inter-group disparities. The LEfSe analysis unearthed 35 taxonomic biomarkers across the four groups, with LDA scores exceeding three and *p*-values < 0.05. As illustrated in Figure 4C,D, the *Spirulina* ^High^ group boasted nearly twice the number of diverse taxa as the Running group, while the *Spirulina* ^Low^ group harbored the lowest taxonomic count. This finding intimates that high concentrations of *Spirulina* modulate certain specific gut microbiota in mice subjected to vigorous exercise. At the genus level, *uncultured_archaeon*, *Lactobacillus*, *unclassified_Clostridia_UCG_014*, and *Muribaculaceae* emerged as the principal differentially expressed microbial communities in the Control, Running, *Spirulina* ^Low^, and *Spirulina* ^High^ groups, respectively (Figure 4D). This observed transformation aligns seamlessly with the preceding analyses.

### 3.5. The Change in Gut Microbiota Is Closely Related to the Tension and Oxidative Stress Indicators Induced by Spirulina

**Figure 5:**
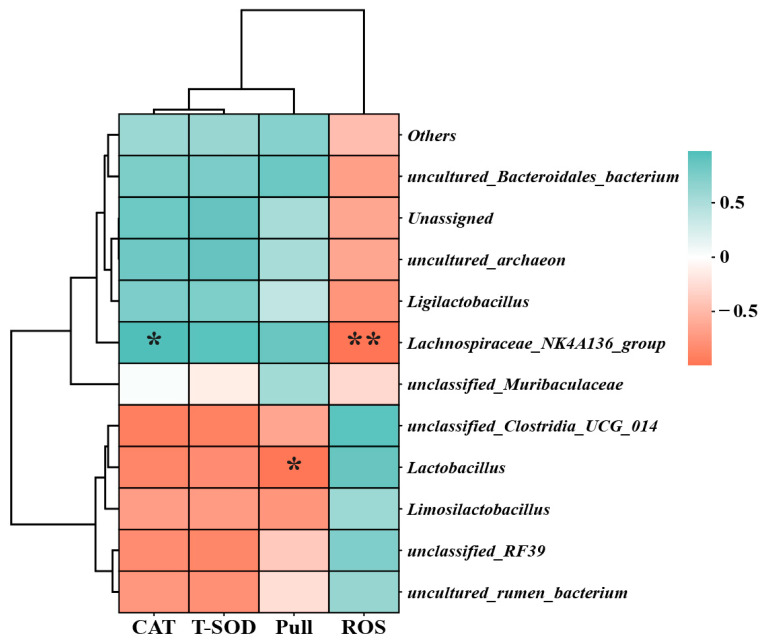
Pearson correlation analysis of different gut bacteria with Pull, CAT, T-SOD, and ROS at the genus level (>1%). The correlation with r > 0.5 or r < 0.5 is indicated by an asterisk. A single asterisk (*) indicates *p* < 0.05, and a pair of star symbols (**) indicates *p* < 0.01.

Given the pronounced alteration in the gut microbiota composition at the genus level instigated by *Spirulina*, it became imperative to ascertain whether such modifications bore a relationship to the tension and oxidative stress indices elicited by *Spirulina*. To address this query, a Pearson correlation analysis was executed, meticulously examining the variations in gut microbiota composition alongside the indicators of tension and oxidative stress. Notably, a significant negative correlation was unveiled between the *Lachnospiraceae_NK4A136_group* and ROS (*p* < 0.01), accompanied by a positive correlation with CAT (*p* < 0.05) (Figure 5). Furthermore, *Lactobacillus* manifested a negative correlation with tensile strength (*p* < 0.05, Figure 5), thus providing valuable insights into the potential interplay between the gut microbiota and the physiological parameters influenced by *Spirulina*.

## 4. Discussion

High-intensity exercise regimens are often associated with the elicitation of adverse sensations during the exercise process and may even engender detrimental impacts on physical well-being [27]. Previous research has unequivocally substantiated that high-intensity resistance exercise holds the potential to attenuate fat mass through the activation of the musculoskeletal system, concomitant with an augmentation of lean body weight and an elevation of the resting metabolic rate [2,27]. Moreover, the investigations conducted by Gavin et al. [28] have conclusively demonstrated that engagement in strenuous dynamic exercises, including running, cycling, rowing, and swimming, precipitates applied physiological stress, which in turn, culminates in insufficient blood perfusion and oxygen delivery to the actively contracting muscles. This physiological state gives rise to a conspicuous disparity between the energy demands of the contracting muscles and their corresponding energy supply, thereby instigating a plethora of disruptions. These encompass impairments in skeletal muscle metabolism, an attenuation of muscle contraction capabilities, and a reduction in strength output, ultimately culminating in an incapacity to sustain exercise velocity or power production, a phenomenon ubiquitously referred to as fatigue.

However, the manifestations of liver and other visceral organ damage consequent to intense exercise are often rather cryptic and frequently evade detection, with the attendant symptoms surfacing only after a protracted latency period. Multiple studies have unequivocally demonstrated that vigorous physical activity is capable of inciting acute liver injury, characterized by aberrant liver morphology and accompanied by oxidative stress and inflammation [29] Additionally, an intricate interconnection exists among exercise, pain, and oxidative stress, which is capable of precipitating fluctuations in oxidative stress levels. For instance, Jolien Hendrix et al. [30] have astutely observed that oxidative stress, exercise, and pain appear to be interwoven through epigenetic mechanisms. They have emphatically underscored that high-intensity and vigorous exercise serves to elevate oxidative stress levels in both trained and untrained individuals, concurrently affecting a reduction in glutathione levels while significantly augmenting MDA concentrations. These findings are in complete concordance with the phenomena manifested in our study, wherein the construction of a high-intensity running training model revealed that four weeks of exhaustive running exercise led to a constellation of adverse outcomes. These encompassed potential sudden diminutions in weight and tension, the infliction of damage upon liver and muscle tissues, the induction of oxidative harm, and perturbations in the diversity and richness of the gut microbiota.

*Spirulina* is regarded as a crucial nutritional supplement. In elite college athletes, the average daily intake of *Spirulina* is 6 g, which is equivalent to 6 g per 60 kg of body weight per day (approximately 100 milligrams per kilogram per day) [17]. In pain research, Mariana et al. have shown that 300 milligrams per kilogram of *Spirulina* holds significant therapeutic potential in the management of inflammatory pain disorders [20]. Consequently, in the current study, we utilized these concentrations to explore the impact of *Spirulina* on high-intensity exercise-induced damage. *Spirulina* has been empirically demonstrated to facilitate convalescence from physical traumas in mice subjected to intense exercise regimens. This salutary influence may be related to the essential nutrients inherent in *Spirulina*, encompassing proteins, vitamins, minerals, and amino acids, which collectively fortify the organism [31] Such a finding is congruent with our results, which suggests that *Spirulina* is capable of augmenting both body weight and grip strength in mice engaged in vigorous physical exertions. Moreover, in the study by Arrari et al., it was mentioned that adding *Spirulina* to drinking water can effectively reduce abdominal fat in chickens and promote growth. This ultimately led to a significant increase in the average weight of chickens, an improvement in their health condition, and an increase in survival rate [32]. Furthermore, the enhancements manifested in the muscle and liver histology of mice treated with *Spirulina* underscore its capacity to preserve the integrity of muscle and liver tissues during periods of strenuous exercise. For illustration, Fatma et al. [32] identified that *Spirulina* is replete with flavonoids and phenolic acids, endowing it with anti-inflammatory, antioxidant, and hepatoprotective properties. Their study divulged that the supplementation of *Spirulina* led to a reduction in liver weight among obese Wistar rats, rectified perturbations in liver redox balance, significantly attenuated liver reactive oxygen species (ROS) production to baseline levels, mitigated fat droplet vacuolization within liver cells, and fostered overall liver health. In addition, Mehdi et al. [33] investigated male rugby league players and ascertained that *Spirulina* supplementation holds the potential to mitigate exercise-induced inflammation and preclude skeletal muscle damage. They also posited that such supplementation could expedite the recuperation of diverse biomarkers, such as F2 Aesop, C-reactive protein (CRP), and creatine kinase (CK).

In our exploration of the impact of *Spirulina* on oxidative stress levels consequent to intense exercise, we unearthed that elevated concentrations of *Spirulina* can conspicuously rectify the oxidative stress disequilibrium precipitated by vigorous physical activity. We postulate that the antioxidant attributes of *Spirulina* may be related to diverse active phytochemicals, like C-phycocyanin β-carotene, and chlorophyll. For instance, studies performed by Yubing Zhang et al. [34] illustrated that phycocyanin could exert antioxidant effects through the gut microbiota of mice via a metabolite axis. Analogously, β-carotene has been shown to alleviate oxidative stress and inflammation induced by cisplatin, resulting in augmented levels of glutathione peroxidase (GSH Px), gamma-glutamyl transpeptidase (GGT), and glutathione (GSH) in this organ (referring to the liver). These compounds may potentiate their antioxidant efficacy by bolstering endogenous antioxidant enzymes and eradicating reactive species, such as superoxide and hydrogen peroxide free radicals. Furthermore, oxidative stress has the capacity to disrupt the equilibrium of the gut flora. Sarmistha Mitra et al. [35] have elucidated that oxidative stress may foster the proliferation of specific pathogenic or pro-inflammatory bacteria while concomitantly diminishing the populations of beneficial or symbiotic bacteria. This discovery aligns with our correlational study, which observed a negative correlation between ROS levels and the *Lachnospiraceae_NK4A136_group*, thereby corroborating the notion that oxidative stress modulates the gut microbiome.

*Spirulina* is ubiquitously recognized as a dietary supplement renowned for its potent antioxidant properties. In clinical investigations, the blue pigment bilirubin C-phycocyanin has been identified as the principal antioxidant constituent of *Spirulina*. For example, it has been corroborated that phycocyanin exhibits antibacterial effects against various pathogens, including *Escherichia coli*, *Klebsiella pneumoniae*, *Pseudomonas aeruginosa*, and *Staphylococcus aureus* [11] This antibacterial activity contributes to explaining why our research indicated that *Spirulina* influences alterations in the gut microbiota ensuing from vigorous exercise. Simultaneously, gut microbiota may exert an influence on oxidative stress levels. In a study by Liu Jiongyan [36] and colleagues, it was discerned that the *Lachnospiraceae_NK4A136_group* was significantly more abundant in the T-L group of broiler chickens. This enrichment was correlated with a diminution in abdominal fat percentage and a positive association with liver total antioxidant capacity (T-AOC) and ileal superoxide dismutase (T-SOD) levels. These findings buttress our correlation study and suggest that the *Lachnospiraceae_NK4A136_group* may play a role in reducing fat and regulating antioxidant activity.

A significant association prevails between exercise and modifications in gut microbiota. During high-intensity exercise, lactic acid levels in muscle tissue increase rapidly. Lactic acid is a product of cell metabolism, and particularly during intense exercise or hypoxia, a large accumulation of lactic acid can lead to muscle fatigue. However, *Lactobacillus* has a stronger tolerance and better adaptability to lactic acid, enabling it to survive better under the intestinal environmental changes caused by high-intensity exercise (such as accelerated blood flow, tissue hypoxia, enhanced transport and absorption capabilities, etc.) [37]. These factors may well account for the significant decrease in the pulling force of mice and the more than 40-fold increase in abundance of Lactobacillus in the running group in our study, which are consistent with the correlation analysis results showing a negative correlation between *Lactobacillus* and tensile strength. Nevertheless, after *Spirulina* intervention, the abundance of *Lactobacillus* decreased, while that of *Muribaculaceae* increased. Studies have shown that *Muribaculaceae* are highly active polysaccharide-degrading bacteria, containing many genes that encode carbohydrate-active enzymes, including glycoside hydrolases, polysaccharide lyases, and so on, which are key enzymes for degrading polysaccharides [38]. *Spirulina* is rich in many polysaccharides, such as *Spirulina* polysaccharides and sulfated polysaccharides. Therefore, we speculated that *Muribaculaceae* could utilize a variety of polysaccharides as carbon sources to provide energy for themselves and had an advantage in the competition for ecological niches, leading to a decrease in the abundance of other bacterial genera, such as *Lactobacillus*. At the same time, *Muribaculaceae* has a positive effect on host health by degrading polysaccharides and producing beneficial metabolites, which may be one of the reasons why *Spirulina* improves liver and muscle injuries induced by high-intensity exercise.

Consequently, it appears that *Spirulina* supplements may bear a robust association with gut microbiota and antioxidant markers collaborating to alleviate the untoward effects of vigorous exercise. However, the research has certain limitations. For example, the sample size is relatively small, the research period is short, and there is a lack of data on the maintenance of the effects of *Spirulina* after stopping treatment. These factors may lead to overlooking the possibility that the observed effect is transient or dependent upon specific experimental conditions. Meanwhile, the study also lacks a comparison between the effects of *Spirulina* without exercise and the combined effects of *Spirulina* and exercise. Therefore, we are currently conducting a two-month clinical trial on *Spirulina* intervention in normal college students and football players (China Clinical Trial Registration Number: ChiCTR2100045524, https://www.chictr.org.cn/ Date is 28 November 2024.). This trial will confirm the universality and long-term validity of the conclusion. Additionally, investigations into the optimal dosage and long-term safety and effects of *Spirulina* supplements are of great significance for applying these findings to practical situations for athletes and other active individuals. Finally, due to considerations of current research focuses and experimental designs, the article did not directly compare the efficacy of *Spirulina* with that of other supplements (such as vitamin C and resveratrol). However, in the future, through specially designed comparative experiments, the similarities and differences in efficacy between *Spirulina* and other supplements can be clarified from multiple aspects, to better realize the potential value of *Spirulina*. In summary, *Spirulina* seemingly constitutes an efficacious natural supplement capable of enhancing bodily resilience and mitigating the adverse impacts of vigorous exercise on oxidative stress and gut flora configuration. These inferences contribute to the burgeoning body of literature advocating for the utilization of natural bioavailable supplements to support athletic performance and recovery.

## 5. Conclusions

In conclusion, our research validates that *Spirulina* may enhance body weight and tension and repair muscle and liver tissue damage in C57BL/6J mice that engage in intense exercise. Additionally, it can rectify the imbalance of oxidative stress after vigorous exercise and exert a regulatory influence on the gut microbiota. Based on these findings, we further confirm that there exists a close association between the alterations in the gut microbiota and physiological parameters such as oxidative stress and tension. Our research results are of profound significance and offer valuable insights, facilitating a better comprehension of the mechanisms through which *Spirulina* elicits similar responses in humans and other animal locomotion populations, thereby providing a solid foundation for further investigations.

## Data Availability

The raw sequencing data generated in this study have been deposited in the NCBI Sequence Read Archive (http://www.ncbi.nim.nih.gov/sra Date is 28 November 2024.) under accession number PRJNA1191674.

## Supplementary Materials

The following supporting information can be downloaded at: https://www.mdpi.com/article/10.3390/nu17020355/s1. Table S1: Mouse feed content.
