# Supplementary material for: Spirulina Supplementation Alleviates Intense Exercise-Induced Damage and Modulates Gut Microbiota in Mice
Source: Nutrients. 2025 Jan 20;17(2):355. doi: 10.3390/nu17020355 (PMC11768171; doi:10.3390/nu17020355)
Supplement: Supplementary file 1 [file nutrients-17-00355-s001.zip › nutrients-3384057-supplementary.pdf]

nutrients MDPI

Spirulina Supplementation Alleviates Intense Exercise-Induced Damage and Modulates Gut Microbiota in Mice

Chunxia Wang [1,2], Yuting Zhang [1,2], Huijuan Liu [1,2], Shuyu Zhang [1,2], Chengyi Ren [1,2], Jiaming Xu [1,2], Juanjuan Chen [1,2], Feng Wang [3], Qiner Qiu [4], Haimin Chen [1,2#], Wei Wu [1,2#]

[1] State Key Laboratory for Managing Biotic and Chemical Threats to the Quality and Safety of Agro-products, Ningbo University, Ningbo, China
[2] Collaborative Innovation Center for Zhejiang Marine High-efficiency and Healthy Aquaculture, Ningbo University, Ningbo, Zhejiang 315211, China.
[3] Department of Laboratory Medicine, The Affiliated Lihuili Hospital, Ningbo University, Ningbo, China
[4] Faculty of Sports Science, Research Academy of Grand Health, Ningbo University, Ningbo 315211, China
* Correspondence: to whom all correspondence should be addressed: chenhaimin@nbu.edu.cn (Haimin Chen); wuwei@nbu.edu.cn (Wei Wu).

nutrients MDPI

Supplementary Tables

Supplementary Table S1. Mouse feed content

Component	Content	Component	Content	Component	Content
Vitamin A	≥7000 IU	Sodium (Na)	≥2 g	Protein	≥180 g
Vitamin D	≥800 IU	Potassium (K)	≥5 g	Fat	≥40 g
Vitamin E	≥61 IU	Magnesium (Mg)	≥2 g	Fibrin	≥50 g
Vitamin K	≥3 mg	Copper (Cu)	≥10 mg	Lysine	≥8.2 g
Vitamin B1	≥8 mg	Iron (Fe)	≥100 mg	Cystine	≥5.3 g
Vitamin B2	≥10 mg	Zinc (Zn)	≥30 mg	Arginine	≥9.9 g
Vitamin B6	≥6 mg	Manganese (Mn)	≥75 mg	Tryptophan	≥1.9 g
Vitamin B12	≥0.02 mg	Iodine (I)	≥0.5 mg	Histidine	≥4.0 g
Biotin	≥0.1 mg	Selenium (Se)	0.1–0.2 mg	Phenylalanine	≥11.0 g
Niacin	≥45 mg	Calcium (Ca)	10–18 g	Threonine	≥6.5 g
Pantothenic	≥17 mg	Phosphorus (P)	6–12 g	Leucine	≥14.4 g
Folate	≥4 mg	Water	≤100 g	Isoleucine	≥7.0 g
Choline	≥125 mg	Crude ash	≤80 g	Valine	≥8.4 g
